# Women's experiences along the ovarian cancer diagnostic pathway in Catalonia: A qualitative study

**DOI:** 10.1111/hex.13681

**Published:** 2022-11-29

**Authors:** Carmen Vela‐Vallespín, Laura Medina‐Perucha, Constanza Jacques‐Aviñó, Núria Codern‐Bové, Meggan Harris, Josep M. Borras, Mercè Marzo‐Castillejo

**Affiliations:** ^1^ Primary Health Care Center Riu Nord i Riu Sud, Catalan Health Institut Barcelona Spain; ^2^ Research Support Unit Metropolitana Nord University Institute for Primary Health Care Research (IDIAP) Jordi Gol, Catalan Health Institute Barcelona Spain; ^3^ Unitat Transversal de la Recerca Fundació Institut Universitari per a la Recerca a l'Atenció Primària de Salut Jordi Gol i Gurina (IDIAPJGol) Barcelona Spain; ^4^ Unitat Transversal de la Recerca Universitat Autònoma de Barcelona Barcelona Spain; ^5^ Department of Nursing Escola Universitària d'Infermeria i Teràpia Ocupacional de Terrassa, Universitat Autònoma Barcelona Barcelona Spain; ^6^ Evaluation and Qualitative Research ÀreaQ Barcelona Spain; ^7^ Independent Researcher Valencia Spain; ^8^ Department Clinical Sciences, Bellvitge Biomedical Research Institute (IDIBELL) University of Barcelona Barcelona Spain; ^9^ Research Support Unit Metropolitana Sud University Institute for Primary Health Care Research IDIAPJordi Gol Barcelona Spain

**Keywords:** early detection, healthcare seeking, ovarian cancer, prehospital care, primary health care, qualitative research, women

## Abstract

**Background:**

Early detection of symptoms and prompt diagnosis of ovarian cancer are considered important avenues for improving patient experiences and outcomes.

**Methods:**

This qualitative study used a phenomenological approach to perform patient interviews, collecting individual accounts of the prediagnostic phase in women diagnosed and treated for ovarian cancer in 2016–2017. Purposive sampling was used to obtain a diverse sample of 24 participants, while thematic content analysis was used to extract themes and subthemes from interview data.

**Results:**

Three themes and nine subthemes were identified. The first theme was women's delay in recognizing symptoms and seeking care, with subthemes on the lack of knowledge about early signs of ovarian cancer, gender‐related barriers and false reassurance from negative test results. A second theme was missed opportunities during healthcare encounters, due to misattribution of women's symptoms by their physicians, underestimation of symptom severity and need for mediation and inadequate tests and/or false negative results. Finally, interviews highlighted the use of resources and alternative healthcare pathways, including complementary/alternative medicines, access to private health care and women's capacity for action and decision‐making (agency) about their health.

**Conclusion:**

Delayed diagnosis of ovarian cancer is rooted in both individual factors (lack of health literacy, reluctance to seek care) and systemic issues (missed opportunities in healthcare encounters, access to timely specialist care). Further research is needed to investigate the extent to which traditional gender roles and socioeconomic inequalities condition women's ability to manage their own health and to interact with health professionals and the health system.

**Patient and Public Contribution:**

In addition to the patient participation during the interviews, one author was a representative of a patient association.

## 1 INTRODUCTION

Ovarian cancer is the eighth most common tumour in Europe and the gynaecological tumour with the highest mortality.^1^ Estimates of age‐standardized 5‐year net survival generally range from 30% to 50%, figures that have held steady over the past two decades.^2^ Tumour stage at diagnosis is an important factor determining the patients' survival, which is threefold higher in women diagnosed at Stage I compared to Stages III–IV. Unfortunately, most women and other people with ovaries are diagnosed with Stage III or Stage IV cancer.

Ovarian cancer develops mainly in women aged 55 years or older. Genetic factors (BRCA mutations) greatly increase the risk,^3^ while other determinants include age, obesity, first pregnancy after age 35 and nulliparity. In contrast, breastfeeding and oral contraceptives have a protective effect, especially the longer the pills are used.^3^

Given the survival benefits of early diagnosis and the absence of any effective screening test for ovarian cancer,^4^ focusing on detecting symptomatic cases as soon as possible may improve the odds of early diagnosis and successful treatment. However, the symptoms of ovarian cancer can vary from person to person, and these can be decisive for diagnosis.^5^ Ovarian cancer most commonly presents as vague and nonspecific abdominopelvic and urinary symptoms, and women often interpret these as normal changes associated with ageing, menopause or stress.^6^, ^7^, ^8^

The Model of Pathways to Treatment is a conceptual framework for understanding diagnostic and treatment pathways in people with symptomatic cancer.^9^ It identifies five key events in the pathway to care: detection of bodily changes, perceived reasons to discuss symptoms with a health care provider, first consultation with a health care provider, diagnosis and start of treatment. The four intervals between these events are defined as the appraisal, help‐seeking, diagnostic and pretreatment intervals. The patient interval, encompassing the appraisal and help‐seeking intervals, is one of the most important sources of diagnostic delay.^10^

Systematic reviews identify symptom knowledge, interpretation of symptoms as cancer‐related, and beliefs about cancer as three (likely universal) predictors of help‐seeking.^10^, ^11^ Individuals with lower literacy and socioeconomic levels often have lower symptom knowledge and more fatalistic beliefs about cancer.^11^ Additionally, gender appears to be an important barrier to help‐seeking and delayed cancer presentation.^11^, ^12^ The World Health Organization (WHO)^13^ points out that gender norms, socialization, roles and differences in power relations contribute to differences in perceiving diseases, in health behaviours and in access to health services. However, the available systematic reviews show that most studies focus on breast cancer, while the evidence for ovarian cancer remains relatively sparse.^10^

The estimated interval from first noticing ovarian cancer symptoms to receiving a diagnosis varies widely by country.^14^ Delays between the first consultation with symptoms and the diagnostic confirmation and treatment initiation are broadly attributed to the general practitioner (GP) and the healthcare system.^15^ The cancer diagnostic process is often complex, involving different levels of care and it varies significantly with different healthcare models.^15^ Gatekeeper systems have been associated with better quality of care but also with longer diagnostic intervals.^16^, ^17^ An audit of 513 women diagnosed with ovarian cancer in 2013–2014 in Catalonia confirms that long diagnostic intervals are also the norm in this setting, but it did not show an impact on 5‐year survival.^18^ Nevertheless, shortening the interval in ovarian cancer diagnosis remains a key goal for improving quality of care, women's experiences and psychological well‐being^19^ and cancer outcomes.^20^, ^21^

In recent years, qualitative research has emerged as a useful method for an in‐depth exploration of the cancer diagnostic pathway. In our setting, few studies have assessed how women experience ovarian cancer before diagnosis. A phenomenological approach offers the opportunity to effectively capture patterns of meaning from their accounts. The aim of our study is to understand women's experiences of ovarian cancer diagnosis and their interactions with the healthcare system to identify avenues for improving care at the prediagnostic stage in people with ovarian cancer in Catalonia.

## 2 METHODS

### 2.1 Study design and setting

To gain a comprehensive insight into women's experiences of the ovarian cancer diagnostic process, a descriptive qualitative exploratory study was conducted using in‐depth, semistructured, individual interviews, underpinned by a phenomenological approach. Phenomenology aims to explain how individuals give meaning to social phenomena through their lived experience, using a rigorous description of experiences and their detailed analysis to understand how these meanings are created.^22^ The present study was conducted according to the criteria for reporting qualitative research (COREQ).^23^

This study was carried out in public‐sector primary health care in Catalonia. The Catalan Health Service is a national health system model. Primary health care is the gatekeeper to specialist services; however, users may directly present to the emergency department and to sexual health and reproductive care centres (known as ASSIR clinics according to the Catalan acronym). The ASSIR clinics, usually located within primary healthcare centres, follow a one‐stop‐shop approach, bringing together family planning, prenatal care and preventive and health promotion activities, as well as diagnosis, treatment and follow‐up of gynaecological pathologies, including cancer. Around 25% of Catalan public health care users also have private health insurance.^24^

### 2.2 Study participants and recruitment

The sampling frame for patients consisted of women diagnosed with primary ovarian cancer in 2016–2017 who had completed the first phase of treatment with a curative intent (cytoreduction plus chemotherapy) in the Catalan public healthcare system. Participation was on a voluntary basis. Purposive sampling was used to ensure discursive diversity of the participants' characteristics: age, educational level, occupation, geographical residence and hospital level.^25^ These characteristics were used to construct 12 participant profiles, and the sample size was estimated at 24 participants, 2 for each discourse profile. A total of 29 women were recruited by general gynaecologists, oncologists and GPs based on their perceived interest. The interviewer called the women, explained the study objectives and researchers' role and set an interview date. Twenty‐four agreed to participate, four did not meet inclusion criteria and one refused due to scheduling conflicts. Data saturation was reached with a sample size of 24 participants.

Women's age ranged from 40 to 77 years. Five had university studies, and 13 had stopped their schooling at the primary level. Fourteen lived in urban areas, while five were from rural areas.

As for their medical history, nine women had a family history of cancer, including one who carried the BRCA mutation. Two thirds of the women had regular gynaecological check‐ups (ASSIR or private) for routine preventive care or for benign pathologies like ovarian cysts, myomas or endometriosis. The diagnostic intervals ranged from 10 days to 12 months. Most were diagnosed in the private setting, seven through their GP and six in the emergency department. See Table 1 for further details on participant characteristics.

**Table 1:** Patient characteristics

Case	Age	Highest education obtained	Residence^a^	Gynaecological history	Gynaecological history	Health service entry point	Months to diagnosis
1	51	University	City	No	No	GP	3
2	51	University	City	Myomas, HPV, regular check‐ups	Sister—ovarian cancer	Gyno (pub.)	3
3	75	University	City	Ovary removal, 1996; check‐ups every 2–3 years	No	GP	2
4	77	Primary	Village	No	Mother died of cancer; sister—ovarian cancer; sister and son—brain cancer	GP	12
5	70	Primary	Village	No	Maternal grandmother—ovarian cancer; paternal side—several cancers	ED	10–12
6	50	Secondary	Village	Ovarian cyst; check‐ups in public healthcare every 6 months	No	Gyno (pub.)	8
7	59	University	Town	Endometriosis, 1990; check‐ups every 2–3 years	Father and brother—died of cancer	ED	2
8	68	Secondary (Year 10)^b^	Town	Myomas, hysterectomy (age 37); priv. check‐ups	No	Gyno (priv.)	2
9	61	Primary	Village	Annual check‐up (priv.)	No	Gyno (priv.)	3
10	62	Primary	City	Check‐up every 3‐4 years	No	GP	9
11	58	Primary	City	No	No	Gyno (priv.)	3
12	40	Primary	Village	Breast cancer	No	GP	1
13	53	Primary	City	Annual check‐up (priv.)	No	Gyno (priv.)	2
14	76	Primary	Town	No	Sister—died breast cancer	ED	3
15	61	Primary	Town	Bi‐annual check‐up	No	ED	4
16	58	Primary	City	Ovarian cysts; annual check‐up (priv.)	No	Gyno (priv.)	1
17	49	Secondary (Year 10)^b^	City	Annual check‐up (priv.); BRCA gene	Paternal grandmother and aunt—ovarian cancer	Gyno (priv.)	7
18	54	Secondary	Town	Annual check‐up (priv.)	No	Gyno (priv.)	5
19	53	Primary	City	No	No	ED	1
20	62	Secondary (Year 10)^b^	City	No	Grandmother—leukaemia; grandmother—breast cancer	GP	2
21	69	Primary	City	Breast cysts, revision every 6 months	Mother—biliary tract cancer	ED	5.5
22	65	Primary	City	No	No	GP	0.5
23	45	Secondary (Year 12)^b^	City	Ovarian cysts, myomas (2003); private check‐ups	No	Gyno (priv.)	0.33
24	59	University	City	Fibrocystic breasts; myomas; private check‐ups; annual ultrasound	Father—died prostate cancer; maternal aunt—breast cancer	Gyno (priv.)	1

Abbreviations: ED, emergency department; GP, general practitioner; HPV, papillomavirus.

^a^ City: pop. > 50,000; town: pop. 10,000–50,000; villages: pop. < 10,000 (2017 census data.^26^

^b^ Mandatory secondary education is to Year 10 (age 16), followed by 2 years of preuniversity studies (to age 18).

### 2.3 Data collection

A semistructured interview guide was developed, comprising an initial section to elicit women's narrative experiences followed by a set of semistructured questions to ensure the collection of basic data around the key points and time intervals defined in the Aarhus Declaration for Early Cancer Diagnosis Research^15^ (Supporting Information: Box 1). The interview questions were discussed within the multidisciplinary research group, which included professionals from primary care, nursing, political science, sociology and epidemiology, plus a patient from the Association of People Affected by Ovarian Cancer (ASACO).

Sociodemographic data, gynaecological history and family history of cancer were collected on recruitment. Two experienced female qualitative methodologists conducted the interviews (N. C. B. and A. C. C.), which took place in early 2017. They were usually in the woman's home to favour a more personal and in‐depth response, with no supervision by clinicians, and they lasted approximately 60 min and were audio recorded.

### 2.4 Data analysis

All interviews were transcribed verbatim and anonymized (N. C. B.). Thematic content analysis was performed to identify, analyse, organize and report the preliminary themes across the data.^27^, ^28^ Interviewing continued until no new themes were identified, and data were considered rich and saturated. One researcher (C. V. V.) verified transcripts against original audio data, and several authors closely examined the data to identify and agree on the key themes (C. V. V., M. M. C., L. M. P., C. J. A.).

Atlas ti software. 7.5.18 was used to import the text file into the software and analyse the data. All other co‐investigators sense‐checked the transcripts to ensure they reflected the research objectives, and the research team discussed the data to develop an initial coding scheme. Through an iterative process and frequent discussions, the research group identified three key themes that addressed women's experiences, staying as close as possible to the source material. The main findings are described and presented along these lines.

### 2.5 Informed consent statement

Before beginning the interviews, participants were given the opportunity to ask questions or voice concerns, and all signed informed consent.

## 3 RESULTS

Three key themes were identified in the analysis: (1) delay in recognizing bodily symptoms as serious and in seeking timely care; (2) missed opportunities for women during healthcare encounters and (3) use of resources and alternative healthcare pathways. These themes encompassed nine subthemes.

### 3.1 Delay in recognizing bodily symptoms as serious and in seeking timely care

#### 3.1.1 Lack of knowledge about early signs and symptoms of ovarian cancer

Most women were unaware of or disregarded the symptoms associated with ovarian cancer, such as abdominal distension, bloating and pressure in the abdomen and pelvis. Only some women with a family history of ovarian cancer were particularly concerned about their symptoms in relation to ovarian cancer.

…and only later did I realise I had the typical symptoms, which is that you eat and feel full right away. (P9)

#### 3.1.2 Gender‐related barriers

Women tended to normalize their symptoms or attribute them to their gender and age or to natural processes such as menopause. Consequently, the response to symptoms, in some cases, included self‐management and or self‐medication, which delayed consultation with health professionals. Some women attributed the symptoms to psychological causes, such as the stress of caring for a sick child or elderly parents, or to the psychological impact of retiring from work.

I thought it was gas and started taking Aerored [a gas remedy]. My belly swelled a little bit, at that time I was very nervous, I was taking care of my mother with Alzheimer's, maybe it was the nerves. (P36)

I started spotting a little, as if it were a period. I didn't think much of it and blamed it on an argument I'd had with my son. (P26)

In many cases, women were used to having abdomino‐pelvic discomfort and tolerated it without going to the doctor, either because they suffered or had suffered from menstrual cramps or in some cases because they had been diagnosed with fibromyalgia. The symptoms that caused the most alarm among women were progressive abdominal distention and postmenopausal bleeding.

I had painful menstrual cramps … I was wearing an intrauterine device, and the periods are very painful and I didn't insist. (P21)

In some cases, women reported waiting a year or more to go to the doctor's office, prioritizing their work activity, presenting to health services only when their symptoms worsened and were severe enough to interfere with daily life.

I went to Portugal for work, when I arrived, I said to myself: you should have gone to the emergency room instead of going on a trip. (P1)

One of the participants, who had suffered from breast cancer and had a young daughter and a sick father, was told by her gynaecologist that there was a high suspicion of malignant ovarian tumour. The patient refused to undergo surgery because she prioritized having another child over confirming the cancer. Despite her doctors' opposition, the patient did not change her mind until her father died and the symptoms became unbearable.

They decided to perform surgery, but I was not ready, and I refused the operation, I said that I wanted to be a mother again and I stayed like that for almost two years. (P15)

Friends and family members of some interviewees advocated for their well‐being and convinced them to seek medical care. The support of friends and family was crucial in validating women's concerns about their symptoms and overcoming their fears, especially embarrassment and fear of cancer.

When the spotting didn't stop, my friends said I had to go to the doctor. (P26)

#### 3.1.3 False reassurance because of negative check‐up

Many women reported undergoing gynaecological examinations through their private health insurance or ASSIR, in some cases to monitor benign gynaecological pathologies (e.g., myomas, endometriosis) and in others for annual or biannual preventive check‐ups. Receiving a negative result in periodic follow‐up tests or a normal result on cervical screening reassured women that they were free of gynaecological disease, and this led them to disregard symptoms and forego consultations with other specialists.

In May, I had an ultrasound and an annual Pap smear … I had an episode of more severe menstrual pain … as if I had a stone in the lower part, and I decided to go to the private urologist. (P21)

### 3.2 Missed opportunities for women during healthcare encounters

#### 3.2.1 Misattribution of women's symptoms by their physicians

Some participants, once they recognized the bodily changes and the need to seek medical help, reported inadequate diagnostic guidance from their primary care physician, who did not even suspect a gynaecological pathology. Several women were repeatedly treated for urinary tract infections. In one case, a woman consulted the ASSIR about her symptoms, and the attending physician considered that the symptoms were due to a yeast infection brought on by antibiotics prescribed for cystitis.

My GP always treated me with antibiotics and never sent me to a specialist, even when I asked for it. At the same time, the reproductive health clinic kept treating me for a yeast infection. (P4)

In one case, the woman's discomfort was even attributed to a depressive disorder, and her doctor prescribed psychotropic drugs.

I couldn't even stand up, couldn't walk, and I went to the GP, and I said, ‘Send me someplace, I'm so sick it's depressing me!’ And he goes and says, ‘Take this for the depression and you'll see how you feel better’. (P4)

#### 3.2.2 Underestimation of severity of symptoms and need for medication

Some women repeatedly consulted their primary care physician for persistent symptoms. They agreed that their GPs did not have time for them or did not take their concerns seriously enough.

I started to swell…. But it didn't hurt, I was just bearing weight, walking and holding on. I went to the doctor and he said I had nothing: ‘Nothing, nothing, you have nothing, it's perfect…’. (P18)

Some women, especially those who were older and less educated, needed their social network's support for health professionals to validate their symptoms and agree to investigate them. In some cases, a family member (especially adult sons) intervened directly, accompanying the women to the health centre, validating their discomfort and insisting on the seriousness of their condition to obtain a referral to secondary care or hospital emergency departments.

My son and daughter came with me and said: ‘Hey, do me a favour and give us a referral to take my mother to the emergency department [to the hospital]’. ‘Ah, but your mother is fine, her belly is fine, blah, blah, blah’. ‘I don't care, I know my mother, and something is wrong’. They gave us the paper and we went to the hospital. (P18)

#### 3.2.3 Inadequate tests and/or false negative results

In one case, a colonoscopy was requested due to recurrent abdominal pain, which of course did not lead to a diagnosis of ovarian cancer. In another case, although a transvaginal ultrasound was requested, the result was interpreted as negative. Such circumstances can clearly prolong diagnostic intervals by providing (temporary) false reassurance despite the persistence of the symptoms.

In May, I had an ultrasound and an annual Pap smear … and I told him [the gynaecologist] again that I had discomfort … he said that everything was fine and that I should calm down. (P21)

### 3.3 Use of resources and alternative healthcare pathways

#### 3.3.1 Use of complementary/alternative medicines

Some young women interpreted their symptoms as ‘normal’, choosing to self‐manage using naturopathic treatments and alternative medicines.

Over the last month I've had a feeling of being full, and I used alternative medicine treatments to clean out my body. (P9)

In one case, a woman with a previous history of cancer, unable to cope with a second neoplasm, and against the advice of health professionals, resorted to alternative medicines to avoid biomedical therapies.

They decided to operate but … I wanted to fight to be a mother. I took other ways, I took alternative therapies, and so I was holding on for two years. (P15)

#### 3.3.2 Access to private health care

Women with private health insurance had regular gynaecological check‐ups, and if any worrisome symptoms appeared, they had direct and rapid access to their usual private specialists. In some cases where the suspected diagnosis was confirmed in private practice, gynaecologists (many of whom combine public and private practice) used their professional networks to streamline referral to a tertiary public hospital for treatment of ovarian cancer.

My son and daughter‐in‐law went to a gynaecologist we know in Barcelona … three days later we went to the hospital and there were three doctors waiting for me in the consultation room. (P4)

In contrast, some women who struggled to get a diagnosis or faced long waiting lists for tests or referrals from their primary care centre opted to go to a private practice on the advice of their children, fully assuming the physicians' fees and the cost of complementary tests. Others, without the means to access private care and in the absence of a response to their health problems from primary care physicians, used the hospital emergency department as a shortcut to quickly access care. On several occasions, this avenue facilitated the process for diagnosing ovarian cancer, but in other cases, the fragmentation of care caused delays and made it even more difficult to suspect cancer.

I went to the doctor almost every week. He wouldn't send me to any specialist, and then I felt so bad that I went to the hospital two or three times. (P4)

#### 3.3.3 Women's capacity for action and decision‐making (agency) about their health

One participant was a university‐educated woman who was comfortable searching for information through the Internet and finding resources through the public health network. After being discharged from the emergency department of the county hospital with a suspicion of ovarian cancer, she adopted a proactive attitude and managed to be seen at the tertiary hospital of her choice.

I found out … and I picked up the phone and made an appointment: ‘It looks like I have ovarian cancer and I would like a visit with a gynaecological oncologist’ … and they gave it to me on the same Thursday. (P9)

However, this was not a common experience. Many women reported that, beyond face‐to‐face consultation with their physicians, they and their families had difficulty navigating the healthcare system due to poor information, for example, in making follow‐up appointments or obtaining diagnostic test results.

…I have been waiting for an ultrasound since August and they haven't called me. (P13)

## 4 DISCUSSION

This qualitative study identified three key themes and nine subthemes. The first theme was women's delay in recognizing bodily symptoms as serious and in seeking timely care, with subthemes on the lack of knowledge about early signs of ovarian cancer, gender‐related barriers, and false reassurance from a negative check‐up. A second theme was missed opportunities during healthcare encounters, due to misattribution of women's symptoms by their physicians, underestimation of symptom severity and need for medication and inadequate tests and/or false negative results. Finally, interviews highlighted the use of resources and alternative healthcare pathways, including the use of complementary/alternative medicines, access to private health care and women's capacity for action and decision‐making (agency) about their health.

### 4.1 Comparison with findings from other studies

#### 4.1.1 Delay in recognizing bodily symptoms as serious and in seeking timely care

Numerous studies have examined factors affecting the length of the appraisal and help‐seeking intervals for cancer in general^12^, ^29^ and ovarian cancer in particular.^30^, ^31^, ^32^, ^33^ Most women in our study expressed a lack of knowledge regarding the symptoms they were experiencing and shared concern that their symptoms had not aroused suspicion earlier, which is largely consistent with the literature.^30^, ^31^, ^32^, ^33^ The presence of abnormal vaginal bleeding is associated with prompt help‐seeking,^18^ while common and sometimes vague symptoms, such as bloating, pelvic or abdominal pain, difficulty eating or feeling full quickly and urgent or frequent urination, did not usually raise any red flags.^6^, ^31^, ^32^ Some studies have highlighted the low level of awareness of ovarian cancer among the general public,^31^, ^32^ suggesting that if women were able to recognize symptoms of ovarian cancer, this might increase their own suspicion of a malignancy and shorten the help‐seeking interval.^32^ As in other studies, our participants struggled to balance specific bodily sensations with aspects of their life‐worlds (individual, social, perceptual and practical experiences) before consulting a medical doctor.^34^ The normalization of initial symptoms acted as a barrier to help‐seeking^32^ and may be explained, in part, by the subtlety and nonspecificity of early signs of ovarian cancer and by the fact that these often coincide with perimenopausal changes. Women would benefit from gaining more knowledge of the disease and confidence in their own observations of bodily changes^12^ through the promotion of body awareness and health literacy.^35^ Our participants had competing responsibilities related to work and to caring for children, grandchildren and elderly parents, which they frequently prioritized over self‐care, a deeply rooted sociocultural issue among women. In this context, as in other studies,^29^, ^30^, ^32^ women demonstrated a high capacity for disregarding bodily changes and tolerating symptoms, which kept them from seeking medical attention until symptoms become severe and impossible to ignore. Help‐seeking for gynaecological cancer symptoms differs from that for other illnesses because of fears associated with embarrassment of the affected body part and with the perception of cancer itself.^12^ Women of all ages often experience anxiety and fear before and during a pelvic examination due to the invasive nature of the procedure.^36^ Prior experiences of gynaecological violence—situations unfortunately often normalized and rendered invisible—could help explain emotional barriers to help‐seeking for some women. However, in our interviews, women did not openly express feelings of shame or embarrassment about undergoing a pelvic examination. In the interviews, only one woman acknowledged fear of cancer and the consequences of treatment, specifically in relation to loss of fertility, which led her to refuse the recommended treatment. Other fears noted in the literature (though not explicitly mentioned by our participants) include fear of change in body image and the sudden arrival of menopause, which can lead to a feeling of loss of female identity, with possible repercussions on their sexual life and that of their partners.^37^ Validation and legitimization of help‐seeking by the media or by friends and family is known to reduce women's concern about being labelled as time‐wasters^12^ and helps them overcome feelings of shame and fear around the disease and its consequences. In contrast to other studies,^30^, ^32^ in our interviews, women did not express concern that their complaints were inappropriate or trivial, suggesting that fears about wasting their doctor's time were not a barrier to seeking help. In our study, as described elsewhere,^38^, ^39^ normal test results contributed to a false sense of security and delay in seeking care. Even when patients underwent routine investigations and appropriate medical check‐ups, ovarian cancer often went undetected. There is a widespread belief that a negative Pap or papillomavirus test result excludes any type of gynaecological tumour; however, screening is only effective for cervical cancer, not for other forms of gynaecological cancer.^32^

#### 4.1.2 Missed opportunities for women during healthcare encounters

On a woman's first presentation with nonspecific abdomino‐pelvic or urinary symptoms, primary care physicians will rarely suspect ovarian cancer because, fortunately, it rarely turns out to be cancer.^5^, ^40^ Many physicians tended to ignore or normalize the symptoms or misattribute them to urological or digestive causes. This misattribution may be explained to some extent by the low incidence of the tumour and hence the lack of previous knowledge and experience, making it imperative to train and sensitize health professionals to be able to recognize and promptly manage ovarian cancer symptoms. However, as confirmed by other studies,^41^ physicians' requests for and interpretation of the information necessary for diagnosis may also be conditioned by stereotypes, prejudices and their preconceived notions regarding women. Specifically, sexism and ageism can negatively impact how health professionals approach the diagnostic process,^41^, ^42^ normalizing symptomatology and hindering optimal assessment and clinical reasoning,^39^ which partly explains the disparity in care.^41^, ^42^ As is the case with some of our interviewees, omission or delay in the diagnosis of ovarian cancer may also be due to the existence of various biases inherent to healthcare practice, for example, anchoring bias (focusing exclusively on a single piece of information), availability bias (relying too much on already known or readily available information) and confirmation bias (tendency to seek information that supports preconceived ideas).^39^ As described elsewhere, these attitudes and practices, together with the lack of knowledge about this cancer and the difficulty of some physicians to overcome communication problems, could affect the initial evaluation of women with ovarian cancer and lead to misdiagnosis.^43^ Some of our patients, in a situation of great vulnerability due to the persistence or recurrence of their symptoms, recounted that their GPs did not recognize or respond to their problems despite repeated care encounters. Health professionals, who have historically been attributed a role of authority within the doctor‐patient relationship, may be reluctant to change their diagnostic orientation. In this situation, some patients turn to their social network, friends and children, preferably male, to validate the severity of their symptoms and obtain appropriate medical care, challenging the power dynamics established around the physician. Avoiding similar situations in daily practice would imply, as suggested by others,^35^ a change in the approach to physician‐patient relationships, facilitating bidirectional communication, interactions based on empathy, respect for the subjective experiences of users and shared decision‐making. For some women in our study, missed opportunities were related to the performance and interpretation of diagnostic tests by practitioners.^38^, ^39^ This can occur when suspicion of cancer is correctly raised but decisions about planned investigations are suboptimal or inadequate. Such scenarios may be more likely for cancers that share common symptoms (e.g., an abdominal symptom is investigated with a colonoscopy that is negative, and this finding is initially interpreted as a ‘diagnostic closure’). This circumstance can clearly prolong the diagnostic interval and represents a missed opportunity for an accurate diagnosis. However, when the correct tests have been performed, but the results are falsely interpreted as negative without adequate backup reassurance or re‐evaluation mechanisms in place, the difficulties around diagnosis are compounded.^39^

#### 4.1.3 Use of resources and alternative healthcare pathways

Several studies have examined the use of alternative/complementary medicines.^44^ For one woman in our study, the use of these treatments was related to the normalization of symptoms and her consequent desire to self‐manage, while another questioned the appropriateness of biomedical treatments and the authority of the doctor to control her health. In the latter case, the woman's personal history of cancer and possibly a limited social network is likely to have conditioned her response. Our participants showed individual differences in their capacity and opportunity to seek alternative diagnostic pathways (mainly through the private healthcare sector), rooted in their socioeconomic conditions and social networks. Women without the means to access private care came into conflict with professionals and the health system when their problems were not addressed. Lack of trust in their referring physicians, as reflected in other studies,^35^ often translates into the ‘transgression’ of established norms within health systems, for example, presenting to the emergency department without a physician's express indication or refusing the prescribed treatment. Although the present study focused on women's experiences during the prediagnostic stage, the challenges of navigating a complex healthcare system also continue through the diagnostic, treatment and survival phases. In addition to aspects related to gender,^45^ we observed differences rooted in health literacy and in how women process information and make decisions about their care, with implications for the patient experience and health disparities.

### 4.2 Strength and limitations

This study focused on the narratives of women diagnosed with ovarian cancer. The experiences described were in a system based on the gatekeeper model, so they may not be generalizable to other populations or healthcare settings. However, the saturation of the sample data was achieved without new issues arising, and this supports the validity of our findings, which could have implications for many other cancers that affect women in settings similar to ours. In addition, the interview script was agreed upon by all members of the research team, including the representative of a patients association (ASACO). Nevertheless, the study has some limitations. We excluded women with very advanced ovarian cancer, whose ill health would have limited their ability to contribute. Moreover, sampling was done without regard to socioeconomic status, comorbidities or race/ethnicity. However, published studies have not found important differences in marginalized people compared to dominant groups.^12^ Health professionals' choices on which patients to invite for interview also introduces a risk of selection bias. Only women who had a good relationship with their current physician at the time of recruitment could participate, even if their previous experiences with other professionals had been unfortunate. Women's narratives, like any experience, are the result of a process of perception and personal interpretation. In addition, as this is a retrospective study, their recall and interpretation of past events may be affected by their subsequent experiences. It is likely that many women in the interview were not conscious of potential psychological barriers (shame, fear, etc.) when first confronted with symptoms. Moreover, we believe that the initial interview script did not sufficiently probe gender‐related issues. Finally, this study took place in the pre‐COVID‐19 period, when the healthcare panorama was markedly different. However, since the main constraints on the system—time for each patient and access to diagnostic tests—have only been exacerbated by the pandemic, we believe our findings are more relevant than ever.^46^

## 5 CONCLUSION

Women with ovarian cancer reported delays in recognizing bodily symptoms, mainly due to lack of knowledge of symptoms and a failure to interpret them as cancer. Competing demands related to work and family appear to be important barriers to timely help‐seeking. Our results support the notion that prediagnostic contact patterns in primary health care may hold missed opportunities to diagnose ovarian cancer. The factors identified in this study can be addressed through individual interventions and community information campaigns, including by providing women with information about the symptoms of ovarian cancer and their individual risk based on their personal or family history, encouraging body literacy and promoting women's confidence in their observations of bodily changes. At the same time, active and empathic listening and respect for women's subjective experiences are essential in healthcare consultations, as is encouraging two‐way communication and shared decisions. Further research is needed to investigate the extent to which traditional gender roles and socioeconomic inequalities condition women's ability to manage their own health and to interact with health professionals and the health system.

## AUTHOR CONTRIBUTIONS

*Conceiving and designing the study*: Mercè Marzo‐Castillejo, Carmen Vela‐Vallespín, Núria Codern‐Bové and Josep M. Borras. *Obtaining funding and ethical approval*: Mercè Marzo‐Castillejo, Carmen Vela‐Vallespín and Josep M. Borras. *Collecting the data*: Núria Codern‐Bové. *Analysing the data*: Núria Codern‐Bové and Carmen Vela‐Vallespín. *Interpreting the data*: Carmen Vela‐Vallespín, Mercè Marzo‐Castillejo, Laura Medina‐Perucha and Constanza Jacques‐Aviñó. *Writing the report*: Carmen Vela‐Vallespín, Mercè Marzo‐Castillejo, Laura Medina‐Perucha, Constanza Jacques‐Aviñó and Meggan Harris. *Revising the report*: Carmen Vela‐Vallespín, Laura Medina‐Perucha, Constanza Jacques‐Aviñó, Núria Codern‐Bové, Meggan Harris, Josep M. Borras and Mercè Marzo‐Castillejo.

## CONFLICT OF INTEREST

The authors declare that they have no conflict of interest.

### ETHICS STATEMENT

This study was conducted in accordance with the Declaration of Helsinki and received ethics approval from the Ethics Committee of the Primary Health Care Research Jordi Gol i Gurina (IDIAPJGol), number P17/088. The confidentiality of the participants is guaranteed under the Organic Law on the Protection of Data of a Personal Nature (03/2018, December 5) and in accordance with the provisions of Regulation (EU) 2016/679 of the European Parliament and Council of 27 April on Data Protection (GDPR) and relevant national legislation.

## Supporting information

Supplementary information.

## Data Availability

The data that support the findings of the study are available from the corresponding authors upon reasonable request.
