# Supplementary material for: Women's experiences along the ovarian cancer diagnostic pathway in Catalonia: A qualitative study
Source: Health Expect. 2022 Nov 29;26(1):476–87. doi: 10.1111/hex.13681 (PMC9854297; doi:10.1111/hex.13681)
Supplement: Supplementary file 1 — Supplementary information. [file HEX-26--s001.docx]

| **Supplementary box 1. Interview guide**   1. **To start, tell me about your situation before you noticed anything wrong.**  - Family/life situation - Place of residence - Activities - Knowledge of ovarian cancer before being diagnosed - Knowledge of someone you know (family, friends, neighbours) with ovarian cancer  1. **What was the process you went through from the time you noticed something until you were diagnosed with cancer?**  - Initial symptoms that prompted you to seek medical care (primary care, sexual and reproductive health clinic, emergency care, or private physician) or starting point (for example, if you were being followed up or monitored for a gynaecological or other pathology, it was an incidental finding, etc.). - Journey/pathway over time inside the public health care system:   - Services or actors that participated in the diagnosis (GP, gynaecologist in sexual health clinic/hospital, other specialists, emergency physicians)   - Referrals, consultations with different specialists due to referrals (e.g. gastroenterologist) or your own decision (e.g. dietician)   - Access to tests   - Interruptions in the pathway: badly oriented tests or referrals not leading to diagnosis   - Communication with professionals, information received   - Social support during the process (networks, need for support) - Arrival to diagnosis (referral to hospital, surgery, diagnostic confirmation   - Information given and media used (informational materials); need for information   - Mode of communication   - Implementation of intervention, treatment (does diagnostic confirmation follow analysis of surgical specimen?)   - Paperwork   - Support at the time of diagnostic confirmation  1. **How do you think the public health care system has responded to your needs during the diagnostic process? Suggestions for improvement.**  - Information needs: about symptoms related to ovarian cancer, the diagnostic process from different agents, etc. - Need for support in the diagnostic process - Accessibility of services, paperwork |
| --- |
